# Evaluating the Authenticity of the Raw-Milk Cheese Fontina (PDO) with Respect to Similar Cheeses

**DOI:** 10.3390/foods10020350

**Published:** 2021-02-07

**Authors:** Luisa Pellegrino, Johannes A. Hogenboom, Veronica Rosi, Paolo D’Incecco

**Affiliations:** Department of Food, Environmental and Nutritional Sciences (DeFENS), University of Milan, via G. Celoria 2, I-20133 Milan, Italy; luisa.pellegrino@unimi.it (L.P.); john.hogenboom@unimi.it (J.A.H.); veronica.rosi@unimi.it (V.R.)

**Keywords:** capillary electrophoresis, cheese authenticity, Fontina, free amino acids, ion mobility spectrometry, PDO cheese, proteolysis, smear ripened cheese, volatile compounds

## Abstract

The implementation of quality assurance schemes for the assessment of PDO food authenticity is an issue involving manufacturers, traders, retailers and consumers. In this respect, reliable analytical methods are needed to integrate paper-trailing information. The feasibility of distinguishing the Italian Fontina PDO cheese from the generic Fontal cheese was preliminarily evaluated on a set of commercial samples by measuring selected parameters (pH, alkaline phosphatase activity, content of copper, volatiles, extent of proteolysis) related to the different manufacturing processes. The relative profile of free amino acids proved to be a promising tool. A new set of 41 samples of Fontina PDO cheese was collected at representative dairies within the recognized production area and analyzed for free amino acids. A chemometric model of Fontina PDO cheese was built based on the mean content and standard deviation of 15 free amino acids. On this basis, all of the PDO samples were correctly identified, whereas all of the Fontal cheeses were recognized as different cheeses.

## 1. Introduction

Fontina is a traditional Italian PDO cheese produced in the Valle D’Aosta region, on the north-west side of the Alps. The manufacturing process is carried out in accordance with the product specification as it is provided by the EU guidelines for PDO registered products [1]. Raw cow’s milk is added with autochthonous strains of *Streptococcus thermophilus* and *Lactococcus lactis* supplied by the Consortium exclusively to Fontina cheese manufacturers [2], and is coagulated at 36 °C with calf rennet. Curd is finely cut and heated up to 46–48 °C under gentle stirring, then transferred into round moulds (30 to 45 cm diameter) and pressed. The cheese wheels are matured on wooden shelves in local caves (5–12 °C and 95% relative humidity) for a minimum of three months. During this period, cheeses are salted by repeated washings with a saturated NaCl solution. This practice promotes the development of a typical microflora on the surface of the cheese which is responsible for the viscous red-brown smear of the rind [3].

Several studies have investigated the composition and evolution of surface microflora of Fontina [3,4] as well as other smear-ripened cheeses [5,6,7]. The complex microbiota of the smear includes both salt-tolerant yeasts and Gram-positive bacteria, with a predominance of *Corynebacterium*, *Brevibacterium*, *Arthrobacter*, *Microbacterium* genera, and contributes to the development of the characteristic sensory properties of this type of cheese. In particular, studies have highlighted the potential role of selected microbial strains isolated from the smear on the basis of their genome characterization [8,9] or the activity of enzymes purified thereof [10]. In contrast, research dedicated to elucidate the actual role of surface microbiota in the biochemical processes of smear cheese ripening is limited. Smear cheeses are generally characterized by a high moisture content, a high pH value and a high ratio of surface area to volume. Based on these features, an ongoing penetration of the surface microflora with a consequent contribution to the ripening of the whole cheese is expected in this type of cheese [11].

Due to the necessary compliance with the traditional manufacturing conditions and the restricted production area, Fontina PDO cheese commands a premium price. In contrast, cheeses similar to Fontina may be produced in large amounts at industrial plants and with almost no restrictions with respect to the origin of milk and technological operations, resulting in lower costs. In particular, “Fontal” is quite similar to Fontina PDO cheese in terms of shape and surface color, and thus may represent a competitor for the authentic product. Fontal is produced with pasteurized milk and using selected starter cultures. Cheeses are salted in brine, and the surface is covered by paraffin or wax to prevent the development of surface microflora and limit water evaporation. Red pigments are usually included in the surface treatment to give the cheese an appearance close to that of the smear-ripened Fontina. Selected cultures of strains able to produce red-orange pigments are commercially available for the surface treatment of cheeses as an alternative. This type of cheese is sometimes labelled as traditional Fontal.

Studies aimed at differentiating Fontina PDO and Fontal cheeses are very few. Pillonel et al. [12] reported a preliminary study based on the stable isotope ratios. However, only three elements (H, C and N) were significantly different between the two cheeses, making the classification model rather weak.

In this work, a preliminary study was aimed at collecting the relevant chemical and biochemical information to explore characteristics of Fontina PDO cheese that may represent actual differences from Fontal. Considering the obtained information, a specific focus was given to the free amino acid composition of a larger number of samples of Fontina with the aim of building a chemometric model based on this parameter and suitable to distinguish this famous PDO cheese from similar products.

## 2. Materials and Methods

### 2.1. Cheese Samples

Commercial samples of Fontina PDO (*n* = 11), Fontal (*n* = 9) and traditional Fontal (*n* = 2) were purchased from 14 different stores in Northern Italy. A slice from the rind to the core, thus representative of the whole cheese, was available for all of the samples. Forty-one samples of Fontina PDO cheese ripened for 90 to 120 days were collected at 15 dairies located in different areas of Valle D’Aosta and were considered as the reference samples for building the chemometric model characterizing Fontina cheese based on their free amino acid composition.

### 2.2. Cheese Sampling and Characterization

The rind layer (5 mm) was carefully removed using a knife and the cheese was finely grated in a domestic grinder, paying particular attention to achieving good homogeneity. The samples were kept at −20 °C until analysis. Commercial cheese samples were analyzed for pH, measured in a dispersion of 10 g of sample in 100 mL of distilled water (pHmeter Crison Basic 20, Hach Lange, Lainate, Italy), and alkaline phosphatase activity (ISO 11816-2|IDF 155-2:2016). The content of copper was determined by inductively coupled plasma mass spectrometry (ICP-MS aurora-M90, Bruker Daltonics, Italy) after acid mineralization (ISO 21424|IDF 243:2018). Analyses were carried out in triplicate.

### 2.3. Analysis of Volatile Compounds

Volatile compounds (VOCs) of commercial cheese samples were determined in duplicate by gas chromatography–ion mobility spectrometry (GC-IMS). The analytical equipment was a FlavourSpec and the integrated VOCAL software was used for data treatment (G.A.S. Gesellschaft für Analytics Sensorsysteme mbH, Dortmund, Germany). The GC column (Fused silica SE-54-CB-0.5, 30 mL, 0.32 mm ID, 0.5 µm FT) (Sigma-Aldrich, Milan, Italy) was a non-bonded column based on a poly (94% methyl/5% phenyl) silicone phase with 1% vinyl. The splitless injector was set at 80 °C and the headspace injection volume was 100 µL. Nitrogen was used as a carrier gas at the flowrate of 30 mL/min and as a drift gas at 250 mL/min. The column was set at 40 °C. The operating conditions described by Gallegos et al. [13] were used with minor changes. In particular, an aliquot of 2 ± 0.1 g of grated cheese was weighted in a 20-mL vial that was immediately sealed with a septum and conditioned at 70 °C for 8 min. Two vials were prepared for each sample.

### 2.4. Analysis of Primary Proteolysis Extent

The degradation of casein fractions was evaluated by capillary zone electrophoresis (CZE) using a P/ACE^TM^ MDQplus equipment (AB Sciex, Milan, Italy) and a hydrophilically coated capillary column DB-WAX126-7012 (50 cm length, 50 µm i.d., 0.05 µm coating, 100 × 800 µm slit opening) (Agilent Technologies, Milan, Italy). The analytical conditions were those described by D’Incecco et al. [14]. Briefly, 1 g of grated cheese was added with 10 mL of sample buffer, kept at room temperature for 4 h, then diluted 1:5 with the same buffer and filtered with a 0.22 µm PVDF membrane filter (Merck, Milan, Italy) immediately before analysis. Separation was carried out with the column temperature set at 45 °C and using a linear gradient from 0 to 30 kV for 4 min and a subsequent step at a constant voltage of 30 kV. The electropherograms were recorded at 214 nm and the normalized peak areas (peak area counts/migration time) were considered. Peak area ratios were calculated between selected casein breakdown fragments and the corresponding parent casein fractions. In particular, the ratios αs1-I-CN/αs1-CN and Σγ-CN/Σβ-CN described by D’Incecco et al. [14] were considered as indicators of the primary proteolysis of αs1- and β-casein fractions, respectively.

### 2.5. Analysis of Free Amino Acids

Both commercial samples and reference Fontina PDO samples were analyzed. Twenty-one free amino acids (FAAs) were determined using a Biochrom 30+ amino acid analyzer (Biochrom Ltd., Cambridge, UK) according to the procedure described by Hogenboom et al. [15] with minor modifications. Briefly, 3 g of grated cheese was added to 40 mL of 0.2 N sodium citrate buffer, homogenized with an UltraTurrax (Ika T25, Sigma Aldrich, Milan, Italy), deproteinated with 7.5% 5-sulfosalicilic acid and filtered through a 0.22 µm membrane filter prior to injection. The chromatographic separation was achieved by a 7-buffer gradient, including the column washing step, and multipoint calibration was used. Analyses were carried out in duplicate.

### 2.6. Statistical Analyses

Ion mobility data derived from the topographic plots obtained by the GC–IMS of each cheese sample were analyzed by principal component analysis (PCA) using the integrated VOCAL software (G.A.S., Dortmund, Germany). Only two components were considered since additional components did not explain data distribution further.

The statistical difference (*t*-test; two-tailed distribution) between the proteolysis ratios of Fontina PDO and Fontal cheeses was evaluated using the SPSS Win 12.0 program (SPSS Inc. IBM Corp., Chicago, IL, USA). Differences at *p* < 0.05 were considered significant.

The content of the 15 most discriminating FAAs (listed below) determined in the reference samples of Fontina PDO was considered to build a chemometric model following the approach proposed by Hogenboom et al. [15]. Based on the individual mean relative content of the selected FAAs, the appropriate FAA profile for Fontina PDO cheese was defined, and deviations from this typical profile were calculated as Z-scores, i.e., the difference between the actual value and the mean content for every single FAA, expressed as the number of standard deviations. Limits for the Z-scores were used to evaluate the commercial samples in this study.

## 3. Results and Discussion

### 3.1. Cheese Characterization

The appearance of cheese rind is one of the attributes influencing the purchasing decision of consumers. Fontina PDO is characterized by a reddish sticky surface, naturally formed by the microflora of the surrounding environment which colonizes the rind during the ripening period [3]. A minor diversity of coloration of smear cheese rind may depend on the interactions between microbial species, pH, NaCl concentration, and temperature [16,17]. By contrast, the rind of Fontal cheese is usually coated with a protective film (paraffin, wax, or varnish) to prevent the growth of surface microflora and colored with red-brown pigments to obtain a standardized external appearance of the cheese regardless maturity. All of the commercial samples of Fontina PDO (*n* = 11) and Fontal (*n* = 9) fitted these characteristics. In addition, two commercial samples labelled as traditional Fontal were considered in this study since their rind was covered by a thin layer of smear similar to that of Fontina. Appearance of the internal structure was rather similar for Fontina PDO and Fontal cheeses, which are both semi-hard pressed cheeses, with the presence of small irregular openings, the most evident feature observed for two samples shown as an example in Figure S1.

Use of raw milk was confirmed for all of the samples of Fontina PDO cheese by the high levels (ranging from 986 to 3615 mU/g) of alkaline phosphatase activity, comparable to data in the literature [12], whereas both Fontal and traditional Fontal cheese samples proved to be produced with pasteurized milk, the residual activity of the enzyme being lower than 10 mU/g (Table 1) [18].

A remarkable difference was observed between the pH values of the two types of cheese, ranging from 6.01 and 6.46 for Fontina PDO and 5.62 and 5.90 for Fontal (Table 1). Considering that the acidification obtained at coagulation and draining should be similar for the two cheeses, the higher pH value in Fontina cheese is likely a consequence of the smear ripening process [19]. Consistently with this observation, the pH values of the traditional Fontal fell within the range of Fontina. In fact, microorganisms that naturally develop at the surface of this type of cheese include yeasts that degrade lactic and citric acids, favoring the neutralization and subsequent development of bacteria populations. Dolci et al. [3] reported that yeasts increased from 10^3^ up to 10^6^ CFU/cm^2^ on the Fontina cheese surface in the first month of ripening, with a parallel increase of pH value from 5.5 up to around 7.6. Values stayed almost unchanged until 90 days of ripening. The pH values we have found in Fontina PDO were slightly lower since they were measured in the inner mass of the cheese after removal of the rind.

Another characteristic trait of Fontina PDO is that it is traditionally manufactured in a round copper vat, although the use of a steel vat is allowed [2]. Based on the content of copper determined in the commercial cheeses, it was outlined that 10 out of the 11 samples of Fontina PDO and none of Fontal were produced in a copper vat (Table 1). The values of copper varied in the range 3.3 to 8.9 µg/g, the variability possibly being due to manufacturing conditions. Several traditional or PDO cooked cheeses are manufactured in copper vats, including Grana Padano, Parmigiano-Reggiano, Comté, Emmental, and other Swiss cheeses. Data from the literature confirm the content of copper in these cheeses to be in the same order of magnitude as those we found in Fontina PDO, i.e., 5 to 10 times higher than in cheeses manufactured in steel vats [12,20,21]. Copper is an essential co-factor in human metabolic pathways, but it also has a role in the ripening process of cheese. This role has been investigated in a few studies and will be discussed later.

### 3.2. Volatile Compounds

**Figure 1:**
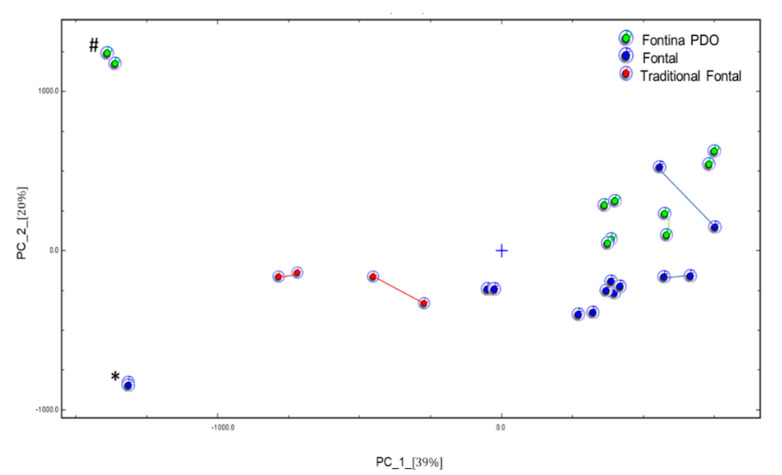
Plot of principal component (PC) analysis of commercial cheese samples. Duplicate samples are connected for clarity in identification. (#) Long ripened Fontina PDO cheese; (*) Fontal cheese produced in a mountain area.

The VOCs have been deeply studied in smear-ripened cheeses since they mostly contribute to the unique aroma profile of this cheese variety [22,23,24]. In particular, the flavor of Fontina PDO proved to be very complex and to include acids, alcohols, esters, aldehydes [25]. In general, raw milk cheeses contain a larger variety of aroma compounds than cheeses produced with pasteurized milk. On this basis, Pillonel et al. [12] achieved a fair distinction of Fontina PDO from Fontal cheese using an electronic nose. In the present study, we have carried out an exploratory attempt to get the same distinction by analyzing the headspace of the cheese samples by GC-IMS. The working principle of this technique is described elsewhere [26] and an example of the obtained topographic plots is shown in Figure S2. Due to a technical problem, not all of the commercial samples could be analyzed. By the non-targeted evaluation of spectral fingerprinting, the PCA model shown in Figure 1 was obtained. The three cheese classes were clustered adequately but with an intermingled sample. In fact, one sample of Fontal cheese fell close to Fontina PDO samples with no apparent explanation, and the two data points of this sample were unexpectedly far from each other, suggesting a poor homogeneity of the sample. With this exception, both Fontal and traditional Fontal samples all fell in the lower part of the plot, whereas Fontina PDO cheeses were positioned in the upper part. The exclusive use of raw milk in Fontina cheese manufacture likely contributes to this relative positioning, as evidenced in a previous study by Gallegos et al. [13]. Adopting the same analytical technique, these authors observed the distinction between goat cheeses produced with raw and pasteurized milk, respectively. Two of our samples fell far from the respective classes: one was a long aged Fontina cheese and the other was a Fontal cheese produced in a mountain area. Although the number of samples was very low, this approach seems to be promising and worthy of further investigation. Due to the high throughput and absence of sample pre-treatment, it could represent a useful screening tool in cheese type distinction.

### 3.3. Primary Proteolysis Extent

**Figure 2:**
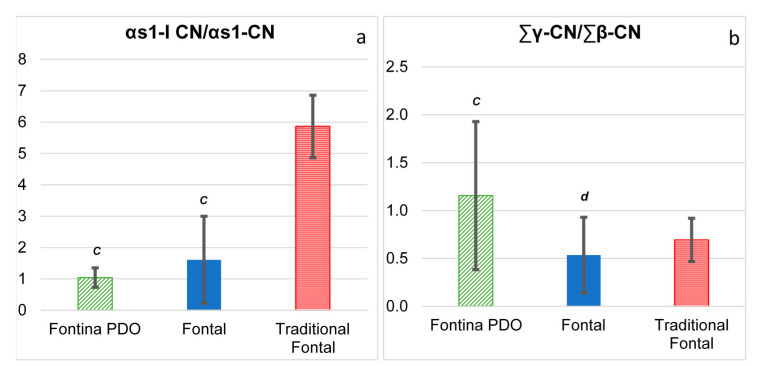
Primary proteolysis extent in commercial samples of Fontina PDO (*n* = 11), Fontal (*n* = 9) and traditional Fontal (*n* = 2) cheeses. (**a**) Degradation of αs1-casein; (**b**) degradation of β-casein. The results are presented as mean ± standard deviation. Statistical analysis was carried out between Fontina PDO and Fontal cheeses only. Mean values with different letters are significantly different (*p* < 0.05; *t*-test).

Initial breakdown of casein in cheese is due to the combined action of coagulant and plasmin. Capillary electrophoresis proved to be an analytical tool capable of high resolution and low-cost analysis of proteins and large peptides and can be successfully adopted to evaluate the extent of primary proteolysis in different types of cheese [14,27,28]. With this aim, the ratio between a casein fragment and the intact parent casein fraction were assumed as indicators of specific proteolytic activities. In particular, chymosin activity was evaluated through the peak area ratio αs1-I-CN/αs1-CN, whereas plasmin activity was evaluated through the peak area ratio γ-CN/β-CN [14]. The ratio αs1-I-CN/αs1-CN was not statistically different between Fontina and Fontal and the values of the latter cheese type fluctuated remarkably, with the highest values reached in traditional Fontal cheese samples (Figure 2a). Accumulation of the αs1-I-CN fragment (f24-199) begins within a few hours after milk coagulation, as a result of the activity of chymosin retained in the curd, and it is largely accepted that chymosin retention mostly depends on the temperature of curd cooking and pH at draining [29]. According to the product specification of Fontina PDO, curd cooking temperature should be within 46 and 48 °C, a rather narrow range, whereas no restrictions are provided for Fontal cheese. Previous papers report that high cooking temperatures lead to an increased inactivation of chymosin and thus to a decreased rate of αs1-casein degradation during cheese ripening [27,30]. Furthermore, the type of coagulant used in cheese making could have had a role. In fact, like most PDO cheeses, Fontina is typically obtained with calf rennet, whereas generic cheeses are nowadays usually obtained with the less expensive microbial or genetic coagulants that have different heat and pH stabilities [31]. Thus, differences in both the cooking temperature and the type of coagulant might account for the high variability of the ratio αs1-I-CN/αs1-CN observed in this study among Fontal cheese samples.

Plasmin has a relatively high heat stability and is not inactivated either by milk pasteurization or curd cooking [29,32]. According to this fact, the hydrolysis of β-casein as the preferential substrate of plasmin continues during ripening and the value of the ratio progressively increases [14,27]. Values of the ratio γ-CN/β-CN were highly fluctuated within each group of cheese, likely due to the different ages of the commercial cheese samples. Despite this variability, the mean value of Fontina samples was significantly higher than that of Fontal samples (Figure 2b).

**Figure 3:**
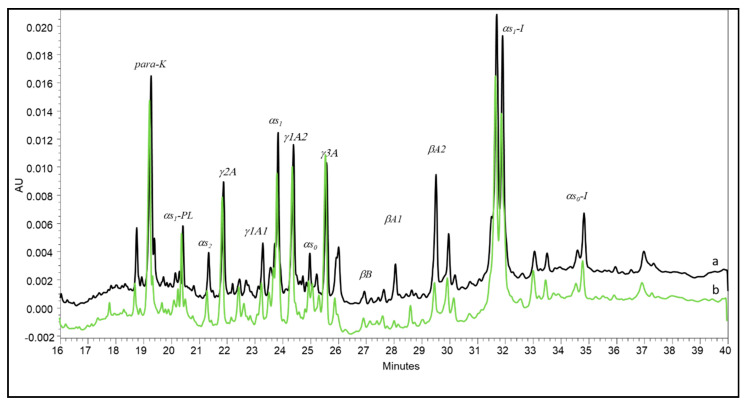
Capillary electrophoresis of Fontina PDO cheeses manufactured in a steel vat (a) and in a copper vat (b) respectively.

Subsequent steps of proteolysis in smear cheeses typically develop much faster in the rind than in the inner cheese body. Hannon et al. [11] adopted stratigraphic sampling to study the proteolysis extent in a smear-ripened farmhouse cheese. The authors observed both αs1- and β-casein to be extensively degraded in the outermost 2 mm-thick layer of the cheese, thus demonstrating the major contribution of enzymes from surface smear microorganisms to casein breakdown in that layer. Due to this evidence, the progress of casein breakdown within smear-ripened cheeses should also be very peculiar compared with that of other cheese types, and this could be a distinctive characteristic of Fontina PDO. However, our results evidenced the difficulty of achieving a reliable characterization of this cheese on the basis of continuously evolving proteolysis indices. Indeed, a number of conditions contribute to shaping the behavior of cheese maturation. Differently, these indices may give evidence to differences within the same cheese types. The CZE pattern of the Fontina PDO sample manufactured in a steel vat had some peculiarities that were evidenced when it was compared with the pattern of a sample manufactured in a copper vat (Figure 3). Interestingly, the two patterns differed almost only for the higher degradation of β-casein in the latter. This difference was previously observed by Pecorari et al. [20] in Parmigiano-Reggiano cheese compared with a cheese manufactured under the same conditions in a steel vat. The role of copper in the ripening of cheeses manufactured in copper vats has been scarcely investigated and is thus controversial. Authors mostly reported its influence on selected properties of bacteria, including the growth rate and proteolytic activity of starter species [33,34] and the germination of *Clostridium* spores [35,36]. To the authors’ knowledge, no data are available on the effect of copper on plasmin activity in cheese, although this aspect is worth further investigation.

### 3.4. Free Amino Acid Profile

The free amino acid (FAA) pattern has been studied in several cheese types, notably raw milk cheeses, proving to be a reliable characterizing parameter [14,22,37]. In the commercial samples of the present research, the total content of FAA ranged from 0.5 to 3.1% of cheese protein in Fontina PDO, and from 0.8 to 6.3% in Fontal and traditional Fontal cheeses, likely as an effect of different ripening periods. Although these ranges overlap, the content of several amino acids, expressed as relative percentage, showed differences between the two cheeses. The most discriminant FAAs between Fontina PDO and Fontal were Ser and Tyr, consistently exhibiting lower values in Fontina than in Fontal, whereas contents of Ile and Lys were significantly higher, but differences could be observed for other FAAs also (Table 2). Previous studies demonstrated that the relative content of single FAAs could be related to cheese age (e.g., Glu, Gln, Lys) [27] or to metabolic pathways of starter or non-starter bacteria (e.g., Arg, Orn, Gaba) [38]. In Fontina PDO, the profile of FAA is shaped by the complex interactions of microbial communities in the raw milk and the smear surface, whereas only bacterial species present in the selected starters added to pasteurized milk are active in Fontal cheese. With this background knowledge, it was decided to analyze a larger number of Fontina PDO samples, collected near a representative number of dairies from the protected production area, in order to build a reliable data set of the FAA composition of this type of cheese. Data obtained for these 41 reference samples (mean values and standard deviations of relative contents) are also shown in Table 2. Based on these data, a chemometric model for Fontina PDO cheese was developed, similarly to the one previously proposed for Grana Padano cheese [15]. First of all, an appropriate FAA profile for Fontina PDO cheese was defined, based on the mean relative content of 15 selected FAAs (see Table 2). Deviations from this typical profile were calculated as Z-scores. In 25 out of the 41 reference samples, all of the calculated Z-scores fell between −2.0 and +2.0; in 11 cases, one score was outside of this range, and in 5 cases, two of the Z-scores were lower than −2.0 or higher than +2.0. The absolute value of Z did not reach a value of 3.0 in any case (Table S1). Therefore, the following chemometric model was proposed for Fontina PDO:

(i) Z-scores for the relative content of the considered FAA must fall between −2.0 and +2.0;
(ii) a maximum number of two Z-scores are allowed to fall between −3.0 and +3.0.

**Figure 4:**
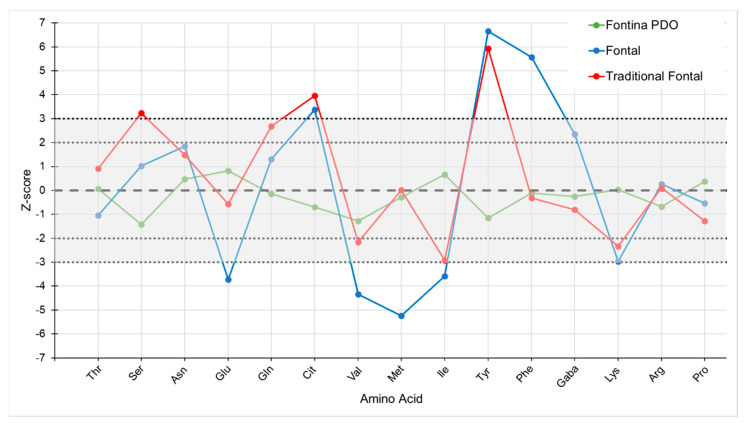
Chemometric model for Fontina PDO cheese based on the profile of 15 free amino acids: behavior of three commercial samples. The grey shaded area identifies the region of authenticity of Fontina PDO cheese.

All commercial samples of Fontina PDO cheese evaluated according to the proposed model were correctly identified, since in none of the samples more than two FAAs presented a Z-score exceeding the value of ±2.0, and in no case the value of ±3.0 was reached. On the contrary, all Fontal cheeses presented two or more FAAs with Z-scores exceeding the value of ±3.0 and even values over 5.0 were observed (Table S2). Hence, it appears possible to distinguish these two cheese types on the basis of their FAA profile. The two samples of traditional Fontal were clearly distinguished from Fontina as well. A graphical representation of this type of evaluation is shown in Figure 4.

The reliability of this model lies in the fact that it considers 15 different variables (i.e., FAAs) at once, and these variables are key compounds originating from proteolytic mechanisms occurring within the cheese during ripening, and are thus related to the intrinsic characteristics and identity of each type of cheese. Furthermore, since the 15 variables are evaluated from the same chromatogram, this approach is well suited for high-throughput analyses. The representativeness of the Fontal cheese class could be further increased, since it is not a defined class; therefore, the sources of this variability might be more than those considered here.

## 4. Conclusions

Fontina PDO and Fontal cheeses share similar external characteristics yet some important differences exist in their origins and cheesemaking processes which are less perceivable by consumers. Aspects of food authenticity, such as the geographical origin of PDO products or specific operations in the manufacture method, need to be evaluated with effective tools since they often identify added-value products. None of the indicators tested in the exploratory study allowed the correct identification of Fontina PDO cheese except the chemometric model based on 15 FAAs. This fingerprinting approach proved to correctly recognize Fontina PDO from similar cheeses and thus to be a promising tool to protect consumers from the misleading labelling of similar cheeses. Additional indicators, such as ALP activity and copper content, could be useful to assess whether the traditional manufacturing process has been correctly followed. Finally, the volatile fraction could provide information about the sensory traits of the PDO cheese. This aspect, however, needs to be further investigated as a possible distinctive parameter.

## Figures and Tables

**Table 1:** Selected parameters (ranges of values) characterizing commercial samples of Fontina PDO, Fontal and traditional Fontal cheeses.

Cheese Type	Samples (*n*)	ALP (mU/g)	pH	Copper (µg/g)
Fontina PDO	11	986–3615	6.01–6.46	3.3–8.9 (0.5) *
Fontal	9	<10	5.62–5.90	0.5–0.8
Traditional Fontal	2	<10	6.13–6.19	0.7–0.8

(*) Out-of-range value found in a sample of Fontina cheese; ALP= alkaline phosphatase activity.

**Table 2:** Free amino acid content (as relative percent) of reference samples of Fontina PDO cheese (*n* = 41) and commercial samples of Fontina PDO (FV), Fontal (FN) and traditional Fontal (FT).

	Asp	Thr	Ser	Asn	Glu	Gln	Gly	Ala	Cit	Val	Met	Ile	Leu	Tyr	Phe	Gaba	Orn	Lys	His	Arg	Pro	Total
Reference Samples																						
Mean	2.37	2.02	1.09	4.21	16.66	1.88	1.62	2.36	0.81	7.29	2.69	4.59	13.93	0.74	8.87	1.79	5.90	12.11	1.77	1.27	5.76	
St Dev	0.93	0.62	0.52	2.12	3.03	1.43	0.29	0.30	0.61	0.65	0.39	0.90	1.35	0.45	1.12	2.21	2.66	1.64	1.21	1.85	1.92	
Commercial Samples																						
FV-01	2.85	1.45	0.28	4.54	13.70	1.40	2.53	3.98	0.33	7.58	2.71	3.79	15.15	0.70	10.71	6.88	8.33	9.87	0.51	0.00	2.71	2.14
FV-02	2.54	2.06	0.35	5.21	19.13	1.67	1.96	2.44	0.39	6.46	2.57	5.18	12.77	0.23	8.75	1.22	6.40	12.15	2.03	0.00	6.50	3.11
FV-03	5.08	2.57	0.56	5.39	7.95	2.51	2.57	2.92	0.72	8.26	2.31	3.64	14.32	1.18	10.88	7.70	8.52	8.93	0.82	0.41	2.77	1.95
FV-04	3.86	1.93	1.52	5.03	9.44	1.72	2.48	3.10	0.76	7.09	2.48	4.06	15.29	0.00	11.5	6.13	11.85	9.02	0.00	0.83	1.93	1.45
FV-05	3.26	2.85	0.32	6.83	20.99	1.49	2.22	2.35	0.63	8.05	3.12	5.79	15.47	0.68	8.05	0.27	2.94	10.22	1.27	0.59	2.62	2.21
FV-06	3.75	2.78	0.60	4.51	18.94	1.97	2.10	3.22	0.52	6.57	2.62	4.11	14.06	0.36	7.98	0.36	6.2	11.64	1.01	0.6	6.08	2.48
FV-07	3.26	2.85	2.20	4.48	18.58	0.65	2.12	2.69	2.16	7.46	3.14	5.13	13.12	1.14	6.76	0.00	3.59	12.51	1.22	0.86	6.07	2.45
FV-08	3.80	1.20	0.54	5.00	15.76	1.52	1.74	4.13	1.63	8.48	2.83	3.04	15.98	1.85	9.46	0.65	2.61	10.43	1.52	4.13	3.70	0.92
FV-09	3.64	1.21	0.40	3.34	12.74	1.92	1.72	3.84	1.11	8.49	2.83	3.64	15.07	0.61	9.71	3.74	4.85	12.64	1.62	1.82	5.06	0.99
FV-10	3.50	1.63	0.75	4.38	18.15	3.38	1.63	2.88	1.25	6.38	2.25	2.13	13.27	1.88	9.01	0.63	3.5	13.64	1.38	4.63	3.75	0.80
FV-11	4.78	1.25	0.83	1.87	12.06	1.66	1.46	4.37	0.83	7.07	2.70	3.74	13.10	1.25	10.4	5.20	5.61	15.38	1.04	1.25	4.16	0.48
FN-01	2.07	3.15	3.15	8.13	14.72	7.07	1.19	2.28	1.19	7.18	2.83	2.67	12.58	3.04	10.99	0.32	4.08	7.07	1.64	2.62	2.04	3.78
FN-02	2.10	3.18	3.13	8.12	15.15	7.01	1.19	2.26	1.27	7.17	2.70	2.72	12.75	3.10	10.95	0.40	4.15	6.71	1.43	2.62	1.89	3.71
FN-03	2.37	1.37	1.62	8.11	5.37	3.75	1.87	2.75	2.87	4.49	0.62	1.37	14.48	3.75	15.11	6.99	7.74	7.24	1.62	1.75	4.74	0.8
FN-04	2.09	1.58	1.96	9.23	16.38	5.00	1.20	2.21	1.64	5.25	1.33	1.45	16.26	3.29	10.44	0.44	6.39	6.39	1.14	2.53	3.80	1.58
FN-05	1.66	3.26	3.24	7.33	12.75	5.89	1.76	2.54	2.75	7.2	3.88	3.50	10.75	3.47	10.24	0.30	2.78	7.19	2.12	1.47	5.93	6.26
FN-06	1.50	2.00	2.63	6.63	11.00	4.00	1.50	3.25	1.88	5.38	1.25	1.38	13.00	3.38	13.75	3.00	8.63	9.25	0.63	2.00	4.00	0.80
FN-07	1.44	2.98	3.15	8.83	4.94	6.23	1.33	2.52	2.70	7.53	2.45	2.00	13.35	3.43	13.20	7.81	4.97	6.37	1.40	1.09	2.28	2.86
FN-08	1.85	3.42	3.52	8.33	14.47	8.58	1.36	2.37	2.79	5.96	2.44	2.27	12.62	3.24	9.03	0.38	4.11	7.08	1.43	1.36	3.38	2.87
FN-09	1.72	2.22	2.78	9.44	7.61	1.89	2.55	3.05	0.33	5.44	1.83	1.78	12.49	1.94	10.94	7.50	0.39	7.83	1.39	12.16	4.72	1.80
FT-01	2.66	2.44	2.31	6.87	12.87	5.30	1.77	3.47	3.63	6.55	3.08	3.27	13.96	3.95	9.41	0.00	4.21	8.35	1.70	0.80	3.40	3.12
FT-02	2.36	2.58	2.76	7.35	14.95	5.71	1.60	2.36	3.24	5.89	2.69	1.96	15.06	3.42	8.51	0.00	5.35	8.26	1.16	1.46	3.31	2.75

## Supplementary Materials

The following are available online at https://www.mdpi.com/2304-8158/10/2/350/s1, Figure S1: Image of commercial samples of Fontina PDO and Fontal cheeses; Figure S2: Topographic plot of a commercial sample of Fontal cheese; Table S1: Z-scores of reference samples; Table S2: Z-scores of commercial samples.
