# Supplementary material for: Evaluating the Authenticity of the Raw-Milk Cheese Fontina (PDO) with Respect to Similar Cheeses
Source: Foods. 2021 Feb 7;10(2):350. doi: 10.3390/foods10020350 (PMC7915116; doi:10.3390/foods10020350)
Supplement: Supplementary file 1 [file foods-10-00350-s001.zip › Figure S2.docx]

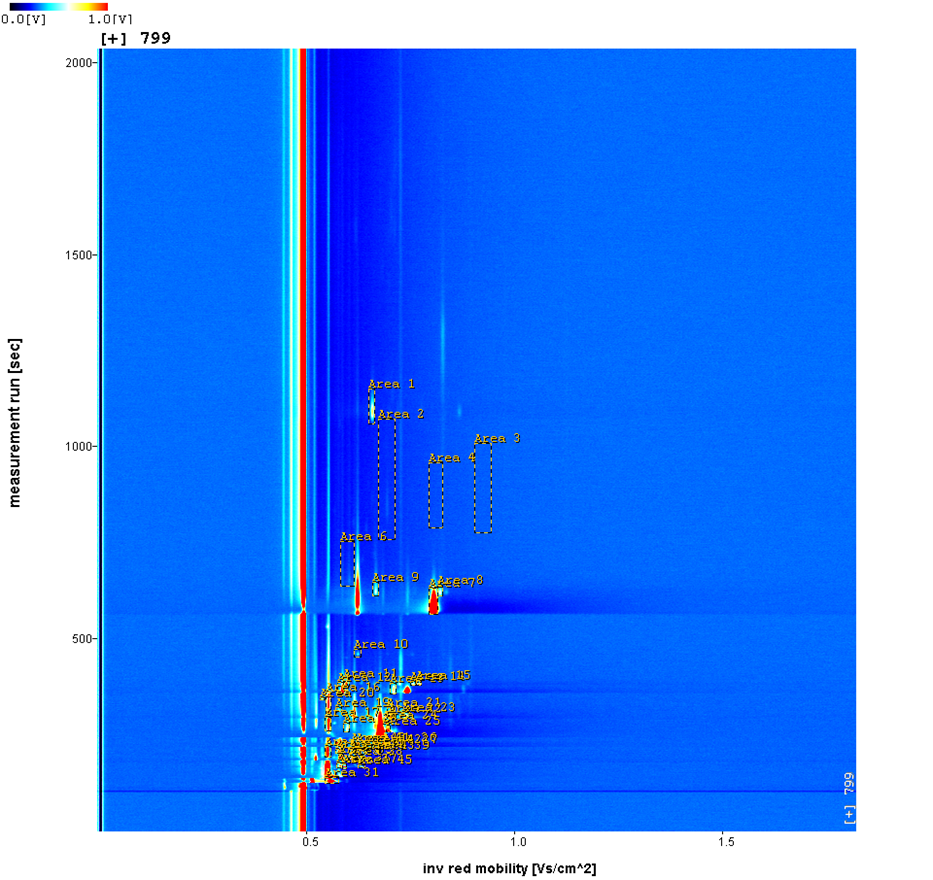


**Figure S2.** Example of topographic plot obtained by gas chromatography-ion mobility spectrometry (GC-IMS) analysis of a Fontal cheese of this study
