# Supplementary material for: Evaluating the Authenticity of the Raw-Milk Cheese Fontina (PDO) with Respect to Similar Cheeses
Source: Foods. 2021 Feb 7;10(2):350. doi: 10.3390/foods10020350 (PMC7915116; doi:10.3390/foods10020350)
Supplement: Supplementary file 1 [file foods-10-00350-s001.zip › Table S1.docx]

**Table S1-** Values of Z-score calculated for reference samples of Fontina PDO (FR) and number of free amino acids (FAA) falling within selected Z-score ranges

| **Reference Sample** | **Thr** | **Ser** | **Asn** | **Glu** | **Gln** | **Cit** | **Val** | **Met** | **Ile** | **Tyr** | **Phe** | **Gaba** | **Lys** | **Arg** | **Pro** | **FAA within**  **Z-score ranges** | |
| --- | --- | --- | --- | --- | --- | --- | --- | --- | --- | --- | --- | --- | --- | --- | --- | --- | --- |
|  |  |  |  |  |  |  |  |  |  |  |  |  |  |  |  | **2<\|Z\|≤3** | **\|Z\|>3** |
| **FR-01** | 0.74 | 1.61 | -0.43 | 0.18 | 0.85 | -0.47 | -0.86 | 0.17 | -0.09 | 1.70 | -1.53 | -0.77 | 0.71 | -0.61 | 1.24 | 0 | 0 |
| **FR-02** | 0.53 | 0.21 | 0.02 | 0.00 | 0.61 | 0.38 | 0.12 | 0.03 | -0.16 | -0.45 | -1.54 | 0.05 | 0.10 | -0.55 | 1.49 | 0 | 0 |
| **FR-03** | 0.75 | 0.77 | 1.38 | -1.67 | **2.12** | -1.32 | -0.60 | 0.32 | -1.35 | -0.92 | -1.95 | 1.37 | 1.94 | -0.69 | -0.50 | 1 | 0 |
| **FR-04** | -0.66 | -0.56 | -0.34 | -0.27 | 1.66 | -0.32 | 0.19 | 1.30 | 0.32 | -0.16 | 1.01 | 0.40 | 0.24 | -0.60 | -0.25 | 0 | 0 |
| **FR-05** | 0.43 | 0.85 | 1.23 | **-2.07** | 1.88 | -0.37 | -0.67 | 0.46 | 0.26 | 1.02 | -0.19 | 1.58 | 0.57 | -0.69 | -1.53 | 1 | 0 |
| **FR-06** | 0.61 | -0.56 | -0.53 | 0.21 | 0.99 | 0.64 | -1.55 | -0.74 | -0.55 | -0.76 | -0.88 | -0.65 | -0.48 | -0.55 | 0.64 | 0 | 0 |
| **FR-07** | 0.83 | -0.49 | -0.50 | 0.20 | -0.19 | **2.16** | -0.03 | 0.86 | -0.32 | -1.05 | -0.55 | -0.41 | 0.51 | -0.69 | -0.25 | 1 | 0 |
| **FR-08** | -0.44 | 0.46 | -1.17 | 0.22 | 0.29 | 0.94 | 0.14 | 0.88 | 0.72 | -0.83 | 1.17 | -0.60 | 1.56 | -0.69 | 0.44 | 0 | 0 |
| **FR-09** | 1.24 | **2.09** | -1.27 | -0.37 | 0.00 | **2.03** | -1.17 | 0.10 | 0.42 | -0.05 | 0.40 | 0.01 | 0.51 | -0.33 | -1.11 | 2 | 0 |
| **FR-10** | 0.50 | -0.18 | 0.45 | 0.19 | 0.31 | 0.25 | 1.01 | -0.05 | 1.24 | -0.27 | -1.28 | -0.31 | 0.61 | -0.69 | 1.18 | 0 | 0 |
| **FR-11** | -0.89 | -1.14 | -1.45 | -0.07 | -0.19 | 0.02 | 1.44 | 0.62 | 0.59 | 0.28 | 0.78 | 0.02 | 0.25 | -0.66 | -1.19 | 0 | 0 |
| **FR-12** | -1.87 | -0.83 | -1.99 | -1.41 | -0.46 | -0.30 | 1.84 | 1.34 | 0.02 | 1.22 | 0.77 | 1.96 | 0.68 | -0.69 | -0.18 | 0 | 0 |
| **FR-13** | 0.40 | 1.16 | 0.45 | -0.42 | 0.24 | 0.53 | 0.54 | 0.00 | 0.22 | 0.62 | -1.00 | -0.11 | 0.15 | -0.53 | 0.59 | 0 | 0 |
| **FR-14** | **2.20** | 1.46 | 0.05 | -0.01 | 0.29 | 0.09 | -0.88 | **2.97** | 1.64 | 0.20 | 1.53 | -0.03 | 0.25 | -0.69 | -1.24 | 2 | 0 |
| **FR-15** | 0.62 | 1.92 | -0.07 | -0.40 | -0.42 | **2.31** | -0.19 | 0.35 | 0.81 | 0.26 | -0.83 | 0.03 | 0.05 | -0.69 | 0.88 | 1 | 0 |
| **FR-16** | -0.27 | 0.50 | -1.57 | 1.07 | -1.06 | 1.10 | 0.69 | -0.37 | 0.60 | -1.29 | -1.18 | -0.59 | 0.95 | 0.79 | 0.78 | 0 | 0 |
| **FR-17** | -0.35 | -0.49 | -1.99 | -0.26 | -1.07 | 1.51 | 0.93 | 0.37 | 0.67 | 0.70 | 0.21 | -0.33 | 1.75 | 0.92 | 1.51 | 0 | 0 |
| **FR-18** | 0.48 | 0.54 | -0.32 | 0.64 | 0.34 | -1.23 | 0.41 | 0.43 | 0.59 | 0.24 | -1.09 | -0.74 | 0.77 | -0.69 | 1.90 | 0 | 0 |
| **FR-19** | 0.31 | 0.89 | -0.53 | 0.02 | -0.13 | 0.94 | 0.88 | 1.43 | 1.16 | -0.25 | 0.42 | -0.43 | 0.26 | -0.67 | 0.74 | 0 | 0 |
| **FR-20** | -0.32 | 0.23 | 0.25 | -0.95 | 1.19 | -1.32 | -0.85 | -0.80 | -1.25 | -0.98 | -0.54 | 0.68 | -1.46 | -0.69 | -0.31 | 0 | 0 |
| **FR-21** | 0.03 | 0.79 | -1.28 | 0.80 | -0.85 | -0.92 | 0.72 | -0.13 | 0.76 | 0.90 | -0.94 | -0.71 | 0.61 | 0.11 | **2.09** | 1 | 0 |
| **FR-22** | 0.82 | -0.91 | -1.99 | 0.48 | -0.90 | 0.11 | 0.77 | 1.38 | 1.01 | -1.32 | 0.07 | 0.11 | 0.79 | 1.38 | 0.88 | 0 | 0 |
| **FR-23** | -0.38 | -0.41 | 0.01 | -0.07 | -1.05 | 0.74 | -0.02 | -0.03 | 0.64 | 1.72 | -0.16 | -0.81 | 0.20 | 0.94 | 1.11 | 0 | 0 |
| **FR-24** | -0.04 | -0.75 | -0.23 | 0.88 | **2.36** | -0.38 | 1.60 | -1.29 | -0.61 | 0.62 | -1.23 | -0.20 | **-2.66** | -0.69 | -0.60 | 2 | 0 |
| **FR-25** | -0.91 | -1.26 | 0.15 | **2.17** | -0.88 | -1.10 | 0.34 | -0.92 | -0.02 | -1.36 | -0.55 | -0.38 | -0.42 | 0.00 | 0.27 | 1 | 0 |
| **FR-26** | -0.22 | 0.13 | -0.13 | -0.99 | 1.85 | -0.27 | -1.24 | -1.03 | -0.91 | 0.75 | -1.00 | 0.57 | -1.41 | -0.69 | 0.55 | 0 | 0 |
| **FR-27** | -0.92 | -0.29 | -0.73 | 0.07 | -0.77 | -1.01 | 0.14 | -0.03 | 0.40 | -0.29 | 0.16 | 0.49 | 1.13 | 0.80 | 0.87 | 0 | 0 |
| **FR-28** | -0.34 | -0.09 | -0.52 | 0.67 | -0.91 | -0.55 | 0.24 | 0.52 | -0.80 | -0.60 | 0.86 | -0.38 | -0.17 | **2.88** | -1.57 | 1 | 0 |
| **FR-29** | 0.04 | -0.15 | 0.45 | -1.16 | **2.30** | -0.28 | **-2.41** | -1.32 | -1.91 | 0.89 | 1.03 | -0.20 | -1.59 | -0.69 | -0.54 | 2 | 0 |
| **FR-30** | -0.43 | -1.06 | -0.11 | -0.20 | -0.77 | -0.73 | -0.33 | -0.45 | -0.97 | -0.96 | 1.08 | -0.12 | 1.15 | 1.77 | -0.25 | 0 | 0 |
| **FR-31** | -0.11 | -0.18 | 0.98 | 0.29 | -0.64 | **2.51** | -0.24 | -0.76 | -1.03 | 0.45 | 0.34 | -0.67 | 0.13 | 1.64 | -0.46 | 1 | 0 |
| **FR-32** | -1.75 | -0.23 | 0.29 | 0.56 | -0.93 | 0.63 | 0.98 | -1.33 | -1.31 | -1.65 | 1.09 | -0.75 | -0.53 | 1.59 | -0.49 | 0 | 0 |
| **FR-33** | -0.36 | 0.91 | 0.81 | 0.92 | -0.61 | -0.34 | 0.72 | 1.10 | 0.36 | **2.06** | -0.03 | -0.43 | 0.61 | -0.69 | -0.28 | 1 | 0 |
| **FR-34** | -1.57 | -1.82 | 0.56 | 0.34 | -1.13 | 0.14 | 1.38 | -1.50 | -0.24 | -1.49 | -0.01 | -0.81 | -0.18 | 0.93 | -0.65 | 0 | 0 |
| **FR-35** | -0.13 | -0.40 | 1.48 | 0.94 | -0.60 | 0.12 | 0.80 | 0.42 | 0.82 | 0.71 | -0.97 | -0.62 | 0.02 | -0.69 | -0.62 | 0 | 0 |
| **FR-36** | 1.49 | 0.48 | 0.85 | 1.86 | -0.86 | -1.17 | **-2.26** | -1.56 | **2.01** | -1.36 | 0.50 | -0.81 | -0.72 | 1.63 | -1.87 | 2 | 0 |
| **FR-37** | 1.21 | 0.58 | **2.26** | 1.35 | -0.19 | -0.51 | -0.42 | 1.11 | 1.92 | 0.02 | -0.08 | -0.65 | -0.09 | -0.69 | -1.38 | 1 | 0 |
| **FR-38** | -0.31 | 0.68 | 1.16 | -0.34 | -0.60 | -1.01 | -1.21 | -1.30 | -1.46 | 0.22 | 0.13 | -0.26 | -1.46 | 0.99 | 0.19 | 0 | 0 |
| **FR-39** | 0.15 | 1.83 | 0.48 | -0.81 | -0.20 | -0.43 | -1.13 | 0.98 | -0.40 | 1.15 | 0.82 | 0.86 | -0.06 | -0.69 | -0.19 | 0 | 0 |
| **FR-40** | -1.09 | -1.38 | 0.91 | 0.03 | -0.89 | -1.10 | -0.52 | -1.63 | -1.55 | -1.07 | 1.67 | -0.49 | -1.19 | **2.19** | 0.23 | 1 | 0 |
| **FR-41** | 0.05 | -0.43 | 1.60 | 0.86 | -0.64 | 0.11 | 1.16 | -0.40 | 0.13 | 1.88 | -0.33 | -0.59 | -0.50 | -0.69 | 0.41 | 0 | 0 |
