# Supplementary material for: Evaluating the Authenticity of the Raw-Milk Cheese Fontina (PDO) with Respect to Similar Cheeses
Source: Foods. 2021 Feb 7;10(2):350. doi: 10.3390/foods10020350 (PMC7915116; doi:10.3390/foods10020350)
Supplement: Supplementary file 1 [file foods-10-00350-s001.zip › Table S2.docx]

**Table S2** – Values of Z-score calculated for commercial samples of Fontina PDO (FV), Fontal (FN) and traditional Fontal (FT) and number of free amino acids (FAA) falling within selected Z-score ranges

|  | **Thr** | **Ser** | **Asn** | **Glu** | **Gln** | **Cit** | **Val** | **Met** | **Ile** | **Tyr** | **Phe** | **Gaba** | **Lys** | **Arg** | **Pro** | **FAA within Z-score ranges** | |
| --- | --- | --- | --- | --- | --- | --- | --- | --- | --- | --- | --- | --- | --- | --- | --- | --- | --- |
| **Samples** |  |  |  |  |  |  |  |  |  |  |  |  |  |  |  | **2.0 <\|Z\|<3.0** | **\|Z\|≥ 3.0** |
| **FV-01** | -0.91 | -1.57 | 0.16 | -0.98 | -0.33 | -0.78 | 0.44 | 0.07 | -0.89 | -0.09 | 1.64 | **2.30** | -1.36 | -0.69 | -1.59 | 1 | 0 |
| **FV-02** | 0.07 | -1.43 | 0.47 | 0.81 | -0.14 | -0.69 | -1.28 | -0.29 | 0.66 | -1.15 | -0.11 | -0.26 | 0.03 | -0.69 | 0.38 | 0 | 0 |
| **FV-03** | 0.88 | -1.02 | 0.56 | **-2.87** | 0.44 | -0.14 | 1.50 | -0.96 | -1.05 | 0.97 | 1.79 | **2.68** | -1.94 | -0.46 | -1.56 | 2 | 0 |
| **FV-04** | -0.14 | 0.82 | 0.39 | **-2.38** | -0.11 | -0.08 | -0.31 | -0.52 | -0.58 | -1.65 | **2.35** | 1.97 | -1.88 | -0.24 | -1.99 | 2 | 0 |
| **FV-05** | 1.34 | -1.5 | 1.24 | 1.43 | -0.27 | -0.28 | 1.33 | 1.11 | 1.34 | -0.15 | -0.73 | -0.69 | -1.15 | -0.37 | -1.63 | 0 | 0 |
| **FV-06** | 1.23 | -0.94 | 0.14 | 0.75 | 0.07 | -0.46 | -0.97 | -0.17 | -0.53 | -0.85 | -0.79 | -0.65 | -0.28 | -0.36 | 0.17 | 0 | 0 |
| **FV-07** | 1.35 | **2.14** | 0.13 | 0.63 | -0.86 | **2.21** | 0.41 | 1.15 | 0.61 | 0.88 | -1.88 | -0.81 | 0.24 | -0.22 | 0.16 | 2 | 0 |
| **FV-08** | -1.32 | -1.06 | 0.37 | -0.30 | -0.25 | 1.34 | 1.84 | 0.36 | -1.72 | **2.45** | 0.53 | -0.52 | -1.02 | 1.55 | -1.08 | 1 | 0 |
| **FV-09** | -1.29 | -1.33 | -0.41 | -1.29 | 0.03 | 0.50 | 1.86 | 0.37 | -1.05 | -0.30 | 0.75 | 0.88 | 0.32 | 0.30 | -0.37 | 0 | 0 |
| **FV-10** | -0.63 | -0.66 | 0.08 | 0.49 | 1.05 | 0.73 | -1.41 | -1.10 | **-2.74** | **2.51** | 0.13 | -0.53 | 0.93 | 1.82 | -1.04 | 2 | 0 |
| **FV-11** | -1.24 | -0.50 | -1.1 | -1.52 | -0.15 | 0.04 | -0.34 | 0.05 | -0.94 | 1.12 | 1.36 | 1.54 | 1.99 | -0.01 | -0.84 | 0 | 0 |
|  |  |  |  |  |  |  |  |  |  |  |  |  |  |  |  |  |  |
| **FN-01** | 1.83 | **3.98** | 1.85 | -0.64 | **3.63** | 0.63 | -0.18 | 0.38 | **-2.13** | **5.1** | 1.89 | -0.67 | **-3.07** | 0.74 | -1.94 | 1 | 3 |
| **FN-02** | 1.88 | **3.94** | 1.85 | -0.50 | **3.58** | 0.75 | -0.18 | 0.03 | **-2.08** | **5.23** | 1.85 | -0.63 | **-3.28** | 0.73 | **-2.02** | 2 | 3 |
| **FN-03** | -1.03 | 1.03 | 1.85 | **-3.73** | 1.30 | **3.37** | **-4.34** | **-5.24** | **-3.58** | **6.65** | **5.57** | **2.36** | **-2.96** | 0.26 | -0.53 | 2 | 3 |
| **FN-04** | -0.70 | 1.68 | **2.38** | -0.09 | **2.18** | 1.37 | **-3.16** | **-3.45** | **-3.49** | **5.64** | 1.40 | -0.61 | **-3.48** | 0.69 | -1.02 | 2 | 4 |
| **FN-05** | **2.00** | **4.16** | 1.48 | -1.29 | **2.80** | **3.17** | -0.14 | **3.04** | -1.21 | **6.04** | 1.22 | -0.67 | **-2.99** | 0.11 | 0.08 | 3 | 2 |
| **FN-06** | -0.03 | **2.97** | 1.14 | -1.87 | 1.48 | 1.74 | **-2.97** | **-3.65** | **-3.58** | **5.83** | **4.36** | 0.55 | -1.74 | 0.40 | -0.92 | 2 | 2 |
| **FN-07** | 1.55 | **3.99** | **2.18** | **-3.87** | **3.04** | **3.08** | 0.37 | -0.59 | **-2.89** | **5.96** | **3.87** | **2.73** | **-3.49** | -0.10 | -1.81 | 3 | 6 |
| **FN-08** | **2.26** | **4.7** | 1.95 | -0.72 | **4.68** | **3.23** | **-2.06** | -0.62 | **-2.58** | **5.54** | 0.15 | -0.64 | **-3.06** | 0.05 | -1.24 | 3 | 2 |
| **FN-09** | 0.33 | **3.26** | **2.47** | **-2.99** | 0.01 | -0.77 | **-2.87** | **-2.17** | **-3.13** | **2.66** | 1.85 | **2.59** | **-2.6** | **5.91** | -0.54 | 7 | 2 |
|  |  |  |  |  |  |  |  |  |  |  |  |  |  |  |  |  |  |
| **FT-01** | 0.68 | **2.36** | 1.26 | -1.25 | **2.39** | **4.6** | -1.15 | 1.01 | -1.46 | **7.11** | 0.48 | -0.81 | **-2.29** | -0.25 | -1.23 | 3 | 0 |
| **FT-02** | 0.91 | **3.24** | 1.48 | -0.57 | **2.68** | **3.96** | **-2.17** | 0.02 | **-2.92** | **5.93** | -0.32 | -0.81 | **-2.34** | 0.10 | -1.28 | 4 | 2 |

Z-score values exceeding ± 2.0 are underlined and bold.
