# Supplementary figures and images for: Evaluating the Authenticity of the Raw-Milk Cheese Fontina (PDO) with Respect to Similar Cheeses
Source: Foods. 2021 Feb 7;10(2):350. doi: 10.3390/foods10020350 (PMC7915116; doi:10.3390/foods10020350)

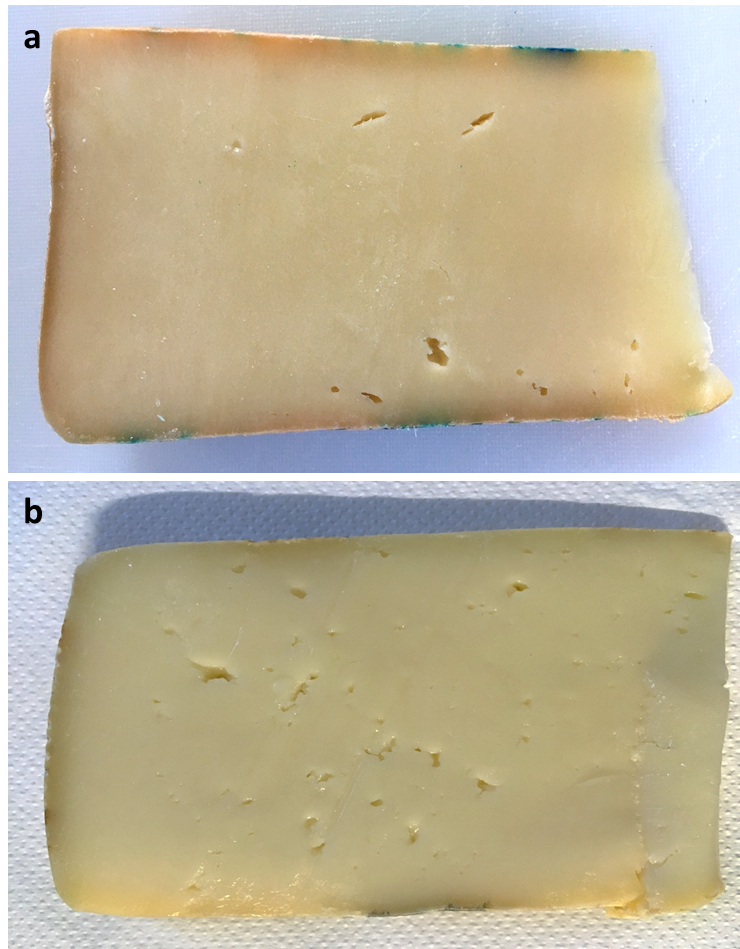


**Figure S1**. Images of Fontina PDO (a) and Fontal (b) cheese slices.

Supplement: Supplementary file 1 [file foods-10-00350-s001.zip › Figure S1.docx]
